# The Need for Demonstrated Clinical Translational Evidence in Submissions to the IEEE Journal of Translational Engineering in Health and Medicine

**DOI:** 10.1109/JTEHM.2026.3680558

**Published:** 2026-05-05

**Authors:** Gerard Boyle, Arturo Forner-Cordero, Anubha Kalra, Vaclav Kremen, Alfonso Maria Ponsiglione, Mahsa Ranji, Sharad Sinha, Xiangyang Tang, Po Yang, Richard B. Reilly

**Affiliations:** Department of Medical Physics and BioengineeringSt. James’s Hospital Dublin D08 NHY1 Ireland; Biomechatronics LaboratoryMechatronics DepartmentCidade Universitária São Paulo 05508-030 Brazil; Auckland University of Technology Auckland 1010 New Zealand; Department of NeurologyMayo Clinic Rochester MN 55905 USA; Department of Electrical Engineering and Information TechnologyUniversity of Naples Federico II 9307 Naples 80125 Italy; Department of Biomedical EngineeringElectrical Engineering and Computer ScienceFlorida Atlantic University 1782 Boca Raton FL 33431 USA; School of Mathematics and Computer ScienceIIT Goa Ponda Goa 403401 India; Department of Radiology and Imaging SciencesEmory University School of Medicine 12239 Atlanta GA 30307 USA; School of Computer Science, Faculty of EngineeringThe University of Sheffield 7315 S1 4DP Sheffield U.K.; School of Medicine, Trinity CollegeThe University of Dublin Dublin D03 X9W9 Ireland

**Keywords:** Translational engineering, clinical translation, clinical validation, clinical endpoints, clinical impact

## Abstract

The IEEE Journal of Translational Engineering in Health and Medicine (JTEHM) exists at the intersection of biomedical engineering and clinical practice. Published articles go beyond laboratory proof-of-concept to provide tangible, real-world evidence of translation into clinical settings. This editorial provides the rationale for manuscripts submitted to IEEE JTEHM to demonstrate evidence of clinical translation. It also provides examples of acceptable forms of evidence and offers guidance to authors on how to meet this expectation. Clinical and Impact—By requiring demonstrated clinical translational evidence IEEE JTEHM endeavours to publish high-quality research with scientific novelty and practical clinical impact. This expectation strengthens the journal’s aim to accelerate the adoption of innovative solutions into healthcare systems and ultimately deliver quantifiable benefits to patients.

## I. Introduction

Translational medical research is defined as bridging the gap between basic medical science research (bench) and clinical research/patient care (bed) [1]. In medicine, engineering solutions, whether wearable technologies, AI-enabled diagnostics, bio-printed implanted systems, or surgical robotics, are only truly translational when validated in clinical settings. While laboratory proofs-of-concept and novel data analysis solutions are essential for improving health, their potential remains unrealised until they can demonstrably improve patient care in real-world clinical settings.

This bridge from bench to bedside is not simply a matter of scaling technology. It requires aligning biomedical engineering innovation with patient needs, clinical workflows, and regulatory requirements. Without rigorous translation, promising biomedical breakthroughs risk failing to progress beyond academically elegant work to clinically validated solutions. Clinical and healthcare environments introduce challenges that no benchtop experiment can fully replicate. This includes challenges such as heterogeneous patient populations, clinical workflow constraints, and the need to integrate seamlessly with existing healthcare infrastructure. Addressing these factors early in the development cycle not only improves the likelihood of clinical adoption but also ensures patient safety and maximises health impact.

By insisting on demonstrated clinical translational evidence, IEEE JTEHM ensures that innovations are evaluated not just for scientific novelty but also for their impact on clinical practice. This approach reinforces the principle that engineering for health is not complete until it is both clinically credible and clinically actionable and thus capable of satisfying the needs of healthcare practitioners, meeting the standards of regulatory authorities, and ultimately delivering quantifiable benefit to patients.

## II. Procedures for Article Submission

The scope of the IEEE JTEHM is to publish articles on advanced engineering technical solutions that have been tested and demonstrated in real-world clinical and healthcare contexts. Without clear evidence of translation, through pilot deployments or clinical trials integrating with healthcare workflows, articles fall outside the journal’s stated scope. For instance, a novel EEG-based algorithm with high detection precision may be scientifically significant, but it lacks clinical utility until it is validated against real-world patient data. To be truly effective, it must demonstrate robust performance within the operational constraints of a hospital’s epilepsy management unit or specialized applications, such as integrated sensing in implantable neuromodulation devices.

### A. Differentiating from Purely Technical Journals

Many scientific journals focus on technical metrics to underscore technical novelty, such as algorithmic accuracy, device sensitivity, or power efficiency. While these are important, IEEE JTEHM distinguishes itself by requiring evidence of applicability in real patient populations under realistic healthcare conditions.

A wearable cardiac monitor may achieve 99% accuracy in arrhythmia detection on simulated or public datasets. However, for submission to IEEE JTEHM, a more robust real-world assessment of the monitor would be expected. Specifically, evidence from ambulatory patient trials where motion artefacts and real-world noise are present. This requirement ensures published articles in IEEE JTEHM report on clinical robustness.

### B. Enhancing Scientific and Clinical Credibility

Clinically tested innovations carry greater weight with healthcare providers, healthcare regulators, and healthcare policymakers. They are also more likely to be cited in clinical guidelines and considered for adoption within hospital systems. For example, researchers reporting a machine learning model that predicts early signs of sepsis gain far more clinical and scientific credibility when they can demonstrate their system has been validated across multiple intensive care units, demonstrating both accuracy and usability for clinicians in time-critical decision-making. Researchers reporting on an AI-based diabetic retinopathy screening tool achieve greater clinical impact when they can demonstrate that the system has been evaluated in community health clinics, proving it can operate under varying lighting conditions, across different imaging devices, and in diverse patient populations.

### C. Focus on Patient-Centred Innovation

Evidence from deployment in clinical settings not only confirms technical performance but also reveals patient and clinician perspectives that may not have become apparent from laboratory testing. A robotic rehabilitation device may perform flawlessly in controlled, lab-based trials, but fail to be adopted in clinical practice if it is cumbersome or time-consuming for therapists to set up or even uncomfortable for patients to use during rehabilitation sessions. Demonstrating clinical evidence ensures that iterative design practices for biomedical engineering solutions are grounded in real feedback, thereby increasing the likelihood of long-term adoption.

## III. Clinical Translational Evidence

Clinical translational evidence is inherently multidimensional and extends beyond laboratory validation. It requires demonstrating that a biomedical engineering innovation has progressed from conceptual or technical feasibility to delivering measurable benefit within real-world clinical settings.

At its core, translational evidence must show that a solution can be effectively integrated into existing clinical workflows and care pathways. This includes demonstrating compatibility with clinical processes, usability by healthcare professionals, and the ability to operate reliably under the constraints of real healthcare environments.

In parallel, evidence of regulatory readiness is essential. Alignment with relevant standards and frameworks (e.g., ISO, IEC, US-FDA, EU-MDR) provides a clear pathway to clinical adoption and ensures that safety, risk management, and usability considerations are appropriately addressed.

Robust translational evidence also requires validation across representative patient populations and care settings. Studies should demonstrate that performance is maintained across diverse clinical conditions, patient demographics, and operational environments, thereby supporting generalisability and scalability.

Most importantly, translational research must demonstrate meaningful impact on patient care. This may include improvements in clinical outcomes, enhanced diagnostic accuracy, increased efficiency of care delivery, or better patient engagement and adherence. Technical performance alone is insufficient unless it is clearly linked to clinically relevant benefit.

In some cases, such as medical education technologies or decision-support tools, direct patient testing may not be required. However, the level of evidence should remain appropriate to the intended use, demonstrating clear utility, user acceptance, and relevance within the clinical context.

The strength of clinical translational evidence can be broadly categorised into three levels:

• **Low-Level Evidence**–Laboratory validation or early feasibility testing, typically involving controlled environments or healthy volunteers. While useful for demonstrating technical feasibility, such evidence does not establish clinical relevance.
• **Moderate-Level Evidence**–Early clinical studies conducted in limited patient cohorts. These studies provide initial insights into usability and clinical applicability but may be constrained by sample size, setting, or generalisability.
• **High-Level Evidence**–Robust clinical validation in real-world settings, often involving multi-centre studies, statistically powered analyses, and alignment with regulatory expectations. This level of evidence demonstrates clinical effectiveness, scalability, and readiness for adoption.

IEEE JTEHM prioritises submissions that demonstrate moderate-to-high clinical translational evidence, with a clear preference for studies that provide strong validation in real-world clinical settings.

## IV. IEEE JTEHM Adopts Technology Readiness Levels (TRLs)

To help authors clarify the level of translation of their studies submitted to IEEE JTEHM, authors are requested to position their work within the framework of Technology Readiness Levels (TRLs), adapted for biomedical and clinical contexts. TRLs provide a structured way to assess how far a technology has progressed from initial concept to widespread clinical adoption. The adapted TRL scale can be summarised as follows:

• TRL 1–2 (Concept and Feasibility)–Basic principles observed, initial hypotheses developed, and laboratory validation in non-clinical settings.
• TRL 3–4 (Prototype Development)–Proof-of-concept demonstrated in the lab, early-stage prototypes tested in controlled environments or with healthy volunteers.
• TRL 5–6 (Preclinical and Early Clinical Validation)–Safety and technical performance demonstrated in relevant environments, early feasibility studies with patient populations.
• TRL 7–8 (Clinical Demonstration and Integration)– Full-scale clinical trials in intended care settings, regulatory submissions underway or completed, integration with clinical workflows.
• TRL 9 (Widespread Clinical Adoption)–Technology deployed in real-world healthcare systems, supported by longitudinal outcome data and post-market surveillance.

IEEE JTEHM is seeking manuscripts which describe work from TRL5–TRL 9. Including the TRL designation in submissions to IEEE JTEHM helps authors position the work in the continuum from discovery to delivery, but also manages reviewers’ expectations.

## V. Acceptable Forms of Translational Evidence for Manuscripts Submitted to IEEE JTEHM

**FIGURE 1.**
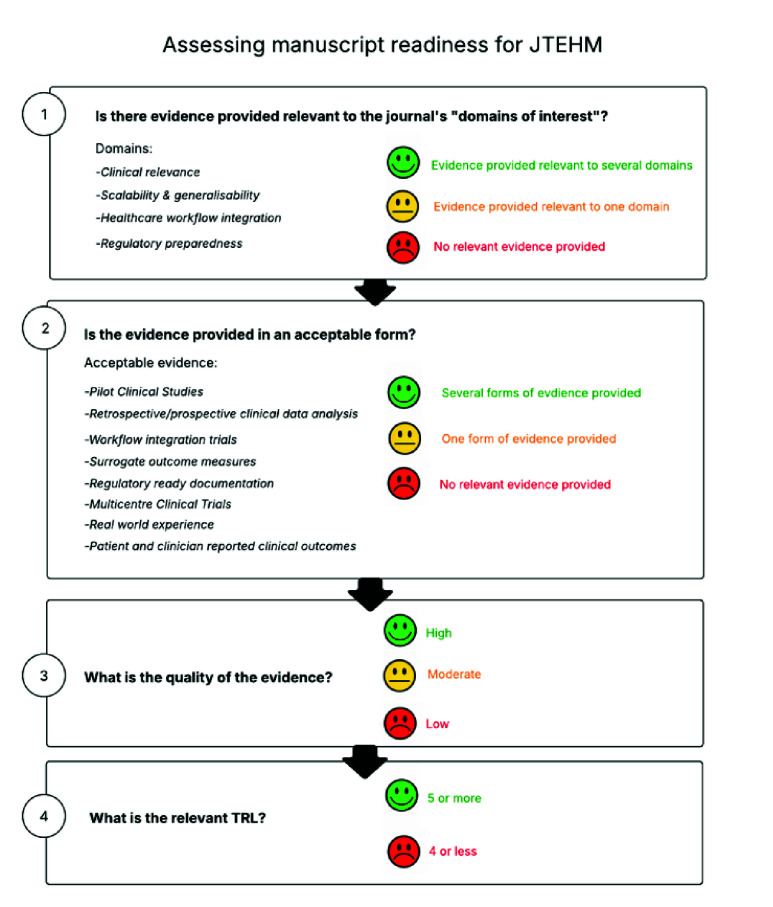
Checklist for assessing a manuscript ahead of submission to IEEE JTEHM.

Translational clinical evidence can be demonstrated through a variety of rigorous methods, each providing unique insights into how well a biomedical innovation performs in real-world clinical environments. IEEE JTEHM recognises that the most cited articles will often integrate multiple forms of evidence, creating a multi-layered picture of technical, clinical, and regulatory readiness. See Fig. 1.

While many submissions to IEEE JTEHM are from authors with an engineering background, others are received from clinical and physician colleagues. While JTEHM’s emphasis is on clinical translation and TRL 5-9, it is not expected that results from a fully randomised clinical trial will be reported. However, they may be desirable in cases where a solution is almost ready to go to the market. Pilot validation studies can be conducted in a clinical setting with institutional review board approval. Such studies typically rely on data collected according to a study protocol, which is then statistically examined for significance.

For example, if a machine-learning-based cuffless blood pressure estimation device has been developed, it can be verified for clinical validity against gold-standard, medical device regulatory-approved cuff-based blood pressure monitoring systems. This is not a “randomised clinical trial” but an examination of how well the machine learning algorithm/model performs against the standard clinical practice in a specific clinical setting. The statistical analysis of such data can help improve the machine learning model and the device design properties, such as its shape, size, and placement. If the machine-learning-based algorithm has been tested only on public datasets, it would be unclear how it would behave when implemented within an appropriate device and used in a clinical setting. Collaborating with engineers and clinicians helps obtain such data and perform clinically relevant analysis.

## VI. Representative Case Examples of Clinical Translation

To clarify the level of clinical translational evidence expected for submission to IEEE JTEHM, we present representative case examples of the types of studies the journal seeks to publish. These examples reflect research that is clinically validated, integrated into real-world workflows, and aligned with regulatory requirements. Authors are encouraged to design studies that demonstrate both technical innovation and measurable clinical impact, ensuring their work contributes meaningfully to improved patient care.

*Case Example: Wearable Gait Sensor for Parkinson’s Disease*

A low-power inertial measurement device worn on the ankle, developed to monitor gait quality and detect freezing episodes in patients with Parkinson’s disease. The device is evaluated in a 100-patient, multi-centre feasibility trial and compared against gold-standard motion capture systems. Results demonstrate that the wearable provides higher sensitivity and specificity for freezing event detection than conventional clinical scoring systems. The project includes workflow testing in both outpatient neurology clinics and home-based physiotherapy settings, with integration into telehealth platforms to support remote monitoring.

*Case Example: Robot-Assisted Surgery Feedback System*

A force-sensing feedback module designed for integration into a commercially available robotic surgical platform. The system provides haptic cues to surgeons to minimise tissue strain and reduce the risk of inadvertent injury during delicate procedures such as cardiac valve repair. In a 50-patient pilot series conducted at two leading cardiac centres, the feedback system reduces tissue-handling errors by 30% compared with surgeries performed without feedback. Surgeon satisfaction scores indicate improved control and reduced fatigue. The technology is supported by usability engineering studies, surgical workflow compatibility assessments, and regulatory pre-submissions to the relevant notified body.

*Case Example: AI-Powered Diabetic Retinopathy Screening Tool*

A cloud-based image analysis platform designed for rapid diabetic retinopathy screening in primary care settings. Trained on over 500 000 retinal images from diverse populations, the system achieves sensitivity and specificity exceeding international screening guidelines. Prospective trials in primary care and hospital clinics demonstrate a significant increase in screening rates and earlier detection of sight-threatening disease. Workflow integration includes compatibility with low-cost fundus cameras and automated reporting to electronic health record systems, ensuring adoption in resource-limited environments.

*Case Example: Remote Cardiac Rehabilitation Platform*

A digital-enabled cardiac rehabilitation solution combining wearable ECG monitoring, adaptive exercise programmes, and live video consultations with physiotherapists. A randomised controlled trial involving 300 post-myocardial infarction patients shows comparable improvements in exercise capacity and quality-of-life scores to those of in-clinic programmes, with significantly higher adherence rates among patients living in rural areas. Integration into national healthcare reimbursement schemes is supported by cost-effectiveness analyses and real-world evidence from post-market registries.

*Case Studies of Systems that Did Not Perform as Expected*

While the example above highlights innovative solutions that have had a positive impact when translated from the ‘bench to bedside’, there are, of course, many examples where this was not the case. There are studies in which, despite excellent results in the laboratory and on simulated clinical data, the solution did not function as predicted when translated to the clinic. IEEE JTEHM is keen to publish such studies. We encourage authors to discuss failures and their causes in relation to biomedical solutions and translational aspects, as a means of educating and informing the scientific and healthcare community.

## VII. Special Considerations for Submissions Employing AI or Machine Learning Methods

For submissions that incorporate artificial intelligence (AI) or machine learning (ML) methods, IEEE JTEHM requires authors to explicitly address the criteria that reviewers will use to evaluate them. This ensures that such studies are assessed not only for technical innovation but also for clinical feasibility, scalability, fairness, and compliance with relevant regulations.

Authors should ensure their manuscripts address the following points in sufficient detail:

1. *Integration into Clinical Workflows*–Describe how the AI/ML solution is embedded into existing clinical processes. Clarify whether it augments, automates, or replaces specific tasks, and provide examples of how it improves or streamlines specific clinical workflows.
2. *Scalability and Clinical Impact*–Discuss the scalability of the solution across different care settings, from small clinics to large hospital networks. Highlight any workflow improvements, efficiency gains, or resource optimisations that have or could result from its deployment.
3. *Representativeness of Training and Validation Data*–Demonstrate that the datasets used for model development and validation are representative of the intended patient population. Address potential biases and outline strategies for handling data drift or distribution changes over time in real-world use.
4. *Validation and Fairness*–Provide evidence that the model has been tested on real patient data or in high-fidelity simulated environments. Include external validation results showing fairness, robustness, and performance across diverse demographic and clinical subgroups.
5. *Resource Requirements and Implementation Challenges*–Detail the cost, infrastructure, and expertise required for implementation. Identify potential barriers to adoption and strategies to mitigate them.
6. *Continuous Monitoring and Interpretability*–Describe processes for monitoring model behaviour in production environments, including ‘dark-production’ phases where features are tested without being fully visible to all users. Discuss interpretability and explainability features to support clinical trust.
7. *Performance Benchmarking and Clinical Utility*–Compare the model’s performance to established baselines or clinical standards. Include measures of clinical utility, such as impacts on workflow efficiency, decision-making quality, or patient outcomes.
8. *Reproducibility*–Provide sufficient methodological detail, including access to code, datasets, and training/evaluation pipelines, to enable reproducibility of results.
9. *Regulatory Considerations*–Discuss relevant medical device regulatory frameworks (e.g., EU MDR, US FDA, HIPAA, GDPR, GxP) and how the study’s design and documentation align with these requirements.

By systematically addressing these points, authors will enable reviewers to assess AI/ML manuscripts fairly and comprehensively and strengthen the likelihood that these innovations will translate successfully into clinical practice.

## VIII. Reviewer’S Perspective–Clinical Reviewer Viewpoint

Evaluating a submission to IEEE JTEHM requires careful consideration from both biomedical engineering and clinical perspectives. Specifically, the clinical reviewer determines whether the innovation has been tested, validated, and presented in a manner that reflects the realities of patient care. The emphasis is not solely on technical performance but on the practical, safe, and meaningful application of that technology within a clinical environment.

Key considerations for a clinical reviewer include:

1. *Clinical Context and Relevance*–Does the submission clearly define the clinical problem being addressed, and is it a priority for patient care or healthcare delivery? Reviewers assess whether the technology targets a well-recognised gap in diagnosis, treatment, monitoring, or workflow efficiency. are the authors citing a challenge published in IEEE JTEHM?
2. *Patient Population and Setting*–Has the technology been tested in a patient population representative of its intended use? Clinical reviewers expect diversity in age, gender, comorbidities, and care settings. A technology validated only in a controlled research environment with volunteers would need evidence from community clinics, emergency departments, or home settings. An innovation that reduces the time to diagnose sepsis would need to contextualise this benefit within existing hospital protocols.
3. *Study Design and Outcomes*–Are the chosen study endpoints meaningful to patient health? Clinical reviewers prioritise outcomes that are directly relevant—such as reduced mortality, faster recovery, or fewer hospital readmissions—over technical metrics unless these are strongly validated. Studies should use appropriate controls, sample sizes, and statistical methods for analysis.
4. *Healthcare Workflow Integration*–Has the technology or solution been integrated into existing clinical workflows? A clinical reviewer will look for evidence of usability testing with actual healthcare staff, compatibility with existing IT systems (e.g., Electronic Health Records), and adaptability across various healthcare settings. Integration challenges that may hinder adoption should be acknowledged and addressed.
5. *Safety and Risk Management*–Have patient safety considerations been fully addressed in realistic clinical environments? A clinical reviewer expects to see data on adverse events, device malfunctions, cybersecurity vulnerabilities, and human factors engineering assessments. Compliance with applicable safety standards is required.
6. *Regulatory Pathway Awareness*–Has the submission demonstrated awareness of the steps required for regulatory approval in the target market(s)? Clinical reviewers will seek evidence of alignment with EU-MDR, US-FDA, or equivalent legislative frameworks to ensure translation to clinical practice.
7. *Evidence of Stakeholder Collaboration*–Was the work conducted in collaboration with clinicians, nurses, allied health professionals, or patients? Submissions that involve end users in the design and evaluation phases are more likely to yield practical, meaningful healthcare solutions.

Ultimately, from a clinical reviewer’s perspective, a successful submission to IEEE JTEHM must demonstrate that the innovation is not only technically sound but also clinically credible, relevant, safe, and ready for implementation in the complex ecosystem of modern healthcare.

## IX. Conclusion

By requiring demonstrated clinical translational evidence, IEEE JTEHM endeavours to publish high-quality research with both scientific merit and practical clinical impact. This expectation strengthens the journal’s aim to accelerate the adoption of innovative solutions in healthcare.
